# A causal model for longitudinal randomised trials with time-dependent non-compliance

**DOI:** 10.1002/sim.6468

**Published:** 2015-03-16

**Authors:** Taeko Becque, Ian R White, Mark Haggard

**Affiliations:** a York Trials Unit, Department of Health Sciences, University of YorkYork, YO10 5DD, U.K.; b MRC Biostatistics Unit, Cambridge Institute of Public Health, School of Clinical Medicine, University Forvie Site, Robinson WayCambridge, CB2 0SR, U.K.; c MRC Multicentre Otitis Media Study Group, Department of Psychology, University of CambridgeDowning Street, Cambridge, CB2 3EB, U.K.

**Keywords:** non-compliance, intention to treat, complier average causal effect, longitudinal model

## Abstract

In the presence of non-compliance, conventional analysis by intention-to-treat provides an unbiased comparison of treatment policies but typically under-estimates treatment efficacy. With all-or-nothing compliance, efficacy may be specified as the complier-average causal effect (CACE), where compliers are those who receive intervention if and only if randomised to it. We extend the CACE approach to model longitudinal data with time-dependent non-compliance, focusing on the situation in which those randomised to control may receive treatment and allowing treatment effects to vary arbitrarily over time. Defining compliance type to be the time of surgical intervention if randomised to control, so that compliers are patients who would not have received treatment at all if they had been randomised to control, we construct a causal model for the multivariate outcome conditional on compliance type and randomised arm. This model is applied to the trial of alternative regimens for glue ear treatment evaluating surgical interventions in childhood ear disease, where outcomes are measured over five time points, and receipt of surgical intervention in the control arm may occur at any time. We fit the models using Markov chain Monte Carlo methods to obtain estimates of the CACE at successive times after receiving the intervention. In this trial, over a half of those randomised to control eventually receive intervention. We find that surgery is more beneficial than control at 6months, with a small but non-significant beneficial effect at 12months. © 2015 The Authors. Statistics in Medicine Published by JohnWiley & Sons Ltd.

## 1 Introduction

Non-compliance or departure from randomised intervention is a common occurrence in randomised controlled trials and can take various forms. For example, some patients randomised to treatment may take too much treatment, too little or none at all. Some participants may switch to another trial intervention or to an intervention outside the trial. In some cases, departures occur after consultation with a physician; in others, they may simply be because of non-adherence. Compliance can both influence and be influenced by the outcome, side effects and other prognostic factors. Intention-to-treat analysis 1,2 has become the standard analysis in the presence of non-compliance as it avoids selection bias and provides an estimate of the effectiveness of a particular programme of treatment. Per-protocol analysis, in which those who adhere to their randomised allocation are compared between randomised arms, is commonly used in addition to intention-to-treat (ITT) analysis. Occasionally, as-treated analysis, where patients are compared according to the intervention received, is also used. Both analyses attempt to measure efficacy but require strong assumptions about the comparability of compliers and non-compliers within randomised arms 3 and are known to be subject to selection bias 4–6.

Instead, we may use a randomisation-based estimate of efficacy, that is, an estimate of a causal effect based on a comparison of randomised arms 3,7. The complier average causal effect (CACE) 8,9 is one such measure of causal effect. The main idea here is to divide the population of interest into several categories or *compliance types*, which specify treatment received under different randomised allocations. Compliance generally refers to treatment received, that is, whether or not the patient received their randomised intervention. Compliance type, on the other hand, is a classification of treatment-received given randomisation and is therefore independent of randomisation. Assuming two randomised arms, treatment and control and assuming compliance is all-or-nothing, that is, individuals either receive all of the treatment or none at all, the possible compliance types are as follows:

1. *Never-takers*: those who never receive treatment regardless of their randomised arm.
2. *Always-takers*: those who always receive treatment regardless of their randomised arm.
3. *Compliers*: those who receive treatment if and only if randomised to treatment, that is, comply with their assignment.
4. *Defiers*: those who receive treatment if and only if randomised to control, that is, do the opposite of their assignment.

Groups of always-takers and defiers are only possible if the treatment is available to those randomised to control. The CACE measures the causal effect of assignment on outcome among the group of compliers. In the principal stratification framework, the compliance types are referred to as *principal strata*, and the CACE is a *principal effect* 10. Compliance types are not fully observable because the behaviour under all possible randomisations cannot be observed for all individuals, but due to randomisation, the expected proportion of patients in each compliance type is the same across randomised arms. Two assumptions, known as exclusion restrictions, are usually made to enable estimation: (1) never-takers have the same mean outcome across randomised arms, and (2) always-takers have the same mean outcome across randomised arms. In addition, it is often assumed that there are no defiers 11.

In this paper, we measure this causal effect as a mean difference, so that the CACE is the difference in mean outcome between compliers randomised to treatment and compliers randomised to control. This CACE may be estimated using instrumental variables (IVs) analysis 12,13. In the context of randomised controlled trials, randomisation is an IV if it affects outcome only through the treatment received. In the simplest setting, the IV estimate of the CACE is the ratio of the ITT effect of randomisation on outcome and the ITT effect of randomisation on treatment received. Under the exclusion restriction and no defiers assumptions, this ratio represents a causal effect of treatment received on outcome 8.

Alternatively, a full probability modelling approach involves specification of a model for the potential outcomes given randomisation and compliance type and allows estimation of the CACE using either maximum likelihood or Bayesian methods 14,15. Maximum likelihood estimation can be performed using the expectation-maximisation algorithm 16, which treats compliance type as unobserved data. The idea is to find the expected compliance type for each individual and to maximise the likelihood to obtain the maximum likelihood estimate of the CACE. The new parameter estimates are then used to calculate the expectation of the missing compliance types. The Bayesian model can be fitted with data augmentation 17, using Markov chain Monte Carlo methods 18.

The same approach may be taken in cases where the data are longitudinal, allowing a time-dependent treatment effect, provided that compliance remains all-or-nothing 19. In trials where the alternative intervention is always available, however, there will be many different compliance patterns, depending on the time at which individuals depart from their allocation. With two or more interventions available at each time, the number of compliance types can quickly become large. An alternative to using all possible compliance types is to use superclasses or latent compliance class principal strata, to summarise longitudinal compliance patterns: ITT contrasts are then made within these superclasses, but these contrasts do not represent causal effects 20. Sitlani *et al.* 21 use a longitudinal structural mixed model (LSMM), an example of a structural-nested model 22, to analyse a surgical trial with non-compliance that is varying over time. They consider a joint model of outcome and treatment, allowing for inclusion of covariates. The average causal effect of treatment is assumed to be a linear function of time. They compare the performance of likelihood-based methods and various semi-parametric methods and state the assumptions required for valid estimation in each case.

In this paper, we propose a causal model for longitudinal data, where intervention group individuals all receive a one-off intervention at the start of the trial, while control group individuals may receive the intervention at any time during the trial. Unlike Sitlani *et al.* 21, we consider the CACE interpretation, generalising the model of 15 by creating a compliance type for each longitudinal pattern of compliance, and we make no assumption about how the treatment effect varies over time: in particular, our model accommodates a transient treatment effect. By jointly modelling outcome and compliance over time, we obtain estimates of the CACE at each time point. We apply this model to data from the trial of alternative regimens for glue ear treatment (TARGET), which compared the effect of a surgical intervention and a control programme on hearing loss in children with otitis media with effusion (‘glue ear’). The surgical intervention was available at all times over the two-year trial, and a large proportion of those randomised to control eventually chose to receive surgery.

In Section 2, we give details of the motivating example along with an ITT analysis. In Section 3, we review existing methods to account for non-compliance, including the standard CACE model. In Section 4, we introduce the CACE model for longitudinal data with time-dependent compliance and various model extensions. In Section 5, we apply these models to the TARGET trial and end with a discussion in Section 6.

## 2 Motivating example

### 2.1 Description of the trial

The TARGET 23 was a UK multi-centre randomised controlled trial that investigated the effect of surgery for children with glue ear. This is a condition in which the middle ear becomes filled with fluid, leading to hearing loss. The trial compared the insertion of ventilation tubes, with and without adenoidectomy, with non-surgical management. The inclusion criteria specified that the children must be aged 3–7years, with no previous ear or adenoid surgery and with greater than 20dB hearing loss in the better ear.

Our analysis includes data from 248 participants: 126 randomised to insertion of ventilation tubes (VT) and 122 randomised to control. The third randomised arm (VT plus adenoidectomy) is ignored for present purposes. VT involved aspiration of fluid remaining in the middle ear, followed by insertion of ventilation tubes in the ear drums. The control arm provided rapid access to antibiotics in the case of resurgent acute infection, although these were rarely used in practice. Improvements in hearing were quantified by hearing level in decibels (dB), with lower measurements indicating better hearing. For this condition, a threshold high value of 40dB represents poor hearing, and less than 15dB is regarded as normal. Other outcomes were also measured, but hearing loss was the main outcome for the power calculation due to its widespread use and the existence of a precise convention on its measurement.

### 2.2 Description of the data

**Figure 1:**
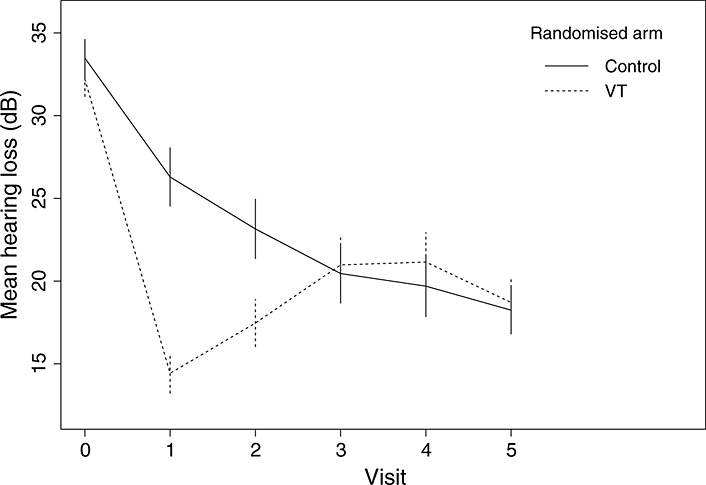
Graph of mean outcome (with 95% confidence intervals) by randomised arm.

Measurements of hearing loss were taken at two pre-randomisation visits, then at 3, 6, 12, 18 and 24months, referred to as post-randomisation visits 1, 2, 3, 4, and 5, respectively ‡. The hearing loss at baseline has mean 33dB and ranges from about 21dB to 46dB. The amount of missing outcome data ranges between about 13% and 18% at each visit, and attrition rates are similar across randomised arms. A descriptive summary of the trial is provided in Table 1. A graph of the observed mean hearing loss against time by randomised arm, along with 95% confidence intervals, is given in Figure 1, following 23. It shows that although VT gives a larger reduction in hearing loss than control by visits 1 and 2, it is comparable to control after visit 3.

**Table 1:** Description of trial of alternative regimens for glue ear treatment (TARGET).

Visit	Months	Randomised to VT (*n*=126)		Randomised to control (*n*=122)		
		*n*	Mean hearing loss (dB)	*n*	Mean hearing loss (dB)	Received VT since previous visit
0	0	126	32.2	122	33.5	0
1	3	109	14.4	106	26.3	13
2	6	106	17.5	105	23.2	20
3	12	110	21.0	100	20.5	17
4	18	103	21.1	98	19.7	13
5	24	108	18.7	102	18.2	3

VT, ventilation tubes.

The published ITT analysis found statistically significant beneficial effects of VT over 3 to 6months but a statistically non-significant negative effect of VT over 12 to 24months. This negative effect ‘occurs because in this period more of the control group have transferred to treatment, and so have functioning VT, than is seen in the surgery groups where VT have mostly fallen out 23’. The present paper aims to correct for such departures, which we now describe in a more detail.

Any child in the VT arm who did not receive their allocated VT and any child in the control arm who received VT were considered to have departed from their randomised intervention. A total of 71 children departed from their allocated intervention, mostly from the control arm to receive surgical intervention (66 children, 54%). Departures from randomised treatment occurred over the duration of the trial, mostly at scheduled visits. The numbers of departures in the control arm between consecutive visits are given in Table 1. Only five of those randomised to VT (4%) received control instead of surgical intervention.

**Figure 2:**
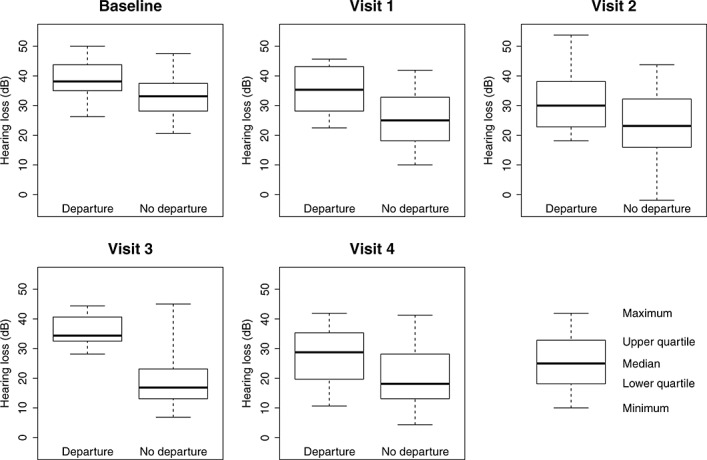
Departures from the control arm. Boxplots of hearing loss for those who depart from control and those who do not depart from control before the next visit.

There were two main reasons for departures in the control arm: early surgical intervention (before visit 1) was mostly due to discontentment with the allocated treatment, whereas later surgical intervention was largely due to deterioration of the child’s condition. To see this, we plot the hearing loss for those randomised to control (Figure 2). At each visit, we compare boxplots of the hearing loss for those who depart from the control arm before the next visit and those who do not depart before the next visit. Those who depart from the control arm tend to have a higher average hearing loss immediately prior to receiving VT than those who have not yet departed from the control arm. In other words, those with worse hearing in the control arm are more likely to depart and receive intervention.

### 2.3 Intention-to-treat analysis of trial of alternative regimens for glue ear treatment data

Let *Y*_*ijk*_ represent the average hearing loss for individual *i* = 1,...,248, visit *j* = 1,2,3,4,5 and allocated treatment *k* = 1,2 (control, VT). An ITT model may be written

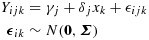
2.1
where *γ*_*j*_ is the mean control arm outcome at visit *j*, *δ*_*j*_ is the treatment effect at visit *j*, *x*_*k*_ is an indicator for treatment being VT (i.e. for *k* = 2) and ***ϵ***_*ik*_ is a vector over *j*. The ITT estimates are given in Table 2.

**Table 2:** Estimates of treatment effect using visits since receiving VT.

Visits since VT	ITT (95% CI)	CACE (95% CI)
1	−11.6(−13.8,−9.3)	−11.6(−14.0,−9.2)
2	−5.6(−8.1,−3.1)	−7.2(−10.1,−4.4)
3	0.8(−1.9,3.5)	−1.4(−4.2,1.4)
4	1.6(−1.3,4.5)	−0.1(−3.5,3.3)
5	0.5(−1.9,2.8)	−0.8(−3.9,2.4)

ITT (intention-to-treat) is the average effect of randomisation on observed outcome at visit *j* (*δ*_*j*_ in model 2.1).

CACE (complier average causal effect) is the average effect of randomisation on outcome at visit *j* in the principal strata of compliers at visit *j* (*β*(*j*) in model 8.

The ITT analysis provides a useful primary analysis of the data and gives estimates of the relative effectiveness of the treatment programmes. However, we may wish to know the efficacy (i.e. causal effect) of the intervention at each time point. Estimation of the causal effect is complicated by the fact that compliance is time-dependent, and the treatment effect itself is also time-varying. In the next section, we look at existing methods to account for non-compliance in randomised trials.

## 3 Accounting for non-compliance

### 3.1 Existing methods

Sitlani *et al.* 21 present an example comparing a surgical intervention with a non-operative treatment, with outcomes measured at five time points after enrollment. They propose a LSMM to account for non-compliance (treatment crossovers) between surgical and non-operative treatment. The LSMM consists of a group average (separated into baseline and time-dependent exposure), subject average (random effects to take into account correlation between measurements on the same individual) and individual observations (error terms assumed to be independent of the random effects). In their model, treatment effect is a linear function of time since receiving surgery, so the model cannot allow for the transient treatment effect that we see in the TARGET trial. The average causal effect at any given time is the difference at that time between the trajectory corresponding to treatment just after enrollment and the trajectory corresponding to no treatment.

Analysis is easiest if treatment depends on baseline characteristics (including randomisation) but not on post-baseline characteristics (an ‘exogenous’ treatment process or ‘no selection’). In practice, treatment often depends on post-baseline characteristics (an ‘endogenous’ treatment process or ‘selection’). Sitlani *et al.* 21 distinguish two types of selection for receiving treatment: direct and indirect. Direct selection depends on covariates observed after baseline but before the time of interest, for example a previous poor outcome leading to a decision to receive surgery. Indirect selection depends on unobserved confounders that affect both treatment and outcome: for example patients with a worse general health condition may elect to receive surgery.

Sitlani *et al.* go on to look at various methods of estimation for the different cases. In the absence of selection, standard tools such as linear mixed effect (LME) model and generalised estimating equations (GEE) may be used. If selection is only direct, the LME and GEE estimators provide consistent estimates of treatment effect provided that the random effect structure is correctly specified. However, if there is indirect selection, LME and GEE estimators that do not explicitly use a selection model can be biased.

Marginal structural models (MSM) enable flexible incorporation of factors that influence treatment timing under marginal modelling assumptions. They require specification of a selection model that includes observed covariates or past treatment that is predictive of treatment. Inverse probability weighting can then be used to obtain consistent estimates of the causal parameters of interest. In order for MSM to be consistent, there must be no unmeasured confounders (no indirect selection), and the form of the selection models must be correctly specified.

G-estimation and IV estimation both aim to be valid under indirect selection by exploiting the randomisation. G-estimation uses the idea that treatment-free potential outcomes for participants randomised to treatment should be on average equal to treatment-free potential outcomes for those randomised to control. This relies on three assumptions: the counterfactual outcomes are independent of randomisation, the structural model is correctly specified and the effect of treatment at a specified time is the same for those who receive it and those who do not (‘no current-treatment interaction’). IV estimators are two stage least squares estimates in which the first equation is a causal model relating outcome and exposure, and the second equation uses an IV, in this case randomisation, to predict exposure. They may be regarded as a special, non-optimal, case of G-estimation. The IV must satisfy the following assumptions in every time period in which a causal effect is to be estimated: (1) random treatment assignment, (2) randomisation affects outcome only via treatment received (exclusion restriction), (3) non-zero average causal effect of randomisation on treatment and (4) those randomised to control and then treated would also have been treated if randomised to treatment (monotonicity, required for the estimates to be interpretable as average treatment effects).

Using simulations, Sitlani *et al.* show that when indirect selection exists, LME, GEE, MSM and G-estimation can be biased, while IV methods tend to avoid bias but are inefficient 21. The bias of their G-estimation appears to arise because the simulation design involved current treatment interaction. They therefore recommend using the joint likelihood of treatment and outcomes in order to obtain efficient and consistent estimates (provided dependence of selection on subject specific latent effects is correctly specified). Estimation may be achieved using Bayesian analysis that explicitly incorporates the selection model. In this paper, we use the joint-likelihood approach via a CACE model to account for indirect selection to treatment. Section 3.2 describes the CACE model that has previously been used for cross-sectional data and longitudinal data where the compliance is binary, and Section 4.1 describes our extension for time-dependent compliance.

### 3.2 Complier average causal effect model for all-or-nothing compliance

We first state the standard CACE model in the simple case of a two arm trial with all-or-nothing compliance and a binary treatment. Let *R*_*i*_ be the randomised arm (*R*_*i*_=1 for treatment and *R*_*i*_=0 for control), and *Y*_*i*_ be the outcome for subject *i*, *i*∈1,...,*n*. Let *D*_*i*_ be an indicator of non-receipt of treatment, so that *D*_*i*_=0 for treated individuals and *D*_*i*_=1 for untreated individuals: this formulation is used as it extends naturally to the time-dependent case in Section 4.1. Let *D*_*i*_(*r*) be the potential value of *D*_*i*_ if subject *i* had been randomised to treatment *r*. Let *Y*_*i*_(*r*,*d*) be the potential outcome for subject *i* if randomised to *r* and treated/untreated according to *d* = 0/1; we only model *Y*_*i*_(0,1) the untreated outcome in the control arm. Let *C*_*i*_ be the latent compliance type for subject *i*, defined in terms of the potential treatments received: individual *i* is an ‘always-taker’, ‘never-taker’, ‘complier’ or ‘defier’ when (*D*_*i*_(0),*D*_*i*_(1)) equals (0,0), (1,1), (1,0) or (0,1) respectively. We allow indirect selection by allowing *C*_*i*_ to be associated with *Y*_*i*_(0,1).

Here, we concentrate on the main form of departure from randomised allocation in TARGET: contamination of the control arm, that is, some of those randomised to control receive treatment. If we assume that those randomised to treatment all receive treatment, then *D*_*i*_(1) = 0 for all *i*, so *C*_*i*_=*D*_*i*_(0): compliance type is treatment received under randomisation to the control arm. Note that in this notation, *C*_*i*_=1 indicates a complier. We do not observe the treatment received under both randomisations for a particular individual so the compliance type *C*_*i*_ is only partially observed. If individual *i* is randomised to control (*R*_*i*_=0) and they receive treatment (*D*_*i*_=0), then they must be an always-taker (*C*_*i*_=0), while if they do not receive treatment (*D*_*i*_=1), then they must be a complier (*C*_*i*_=1). However, if individual *i* is randomised to treatment (*R*_*i*_=1), then they must receive treatment (*D*_*i*_=0), and so they may be either an always-taker or a complier. Therefore, the compliance type *C*_*i*_ is unobserved for those randomised to treatment *R*_*i*_=1.

We assume the causal model

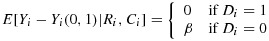
3.1
where *D*_*i*_=*C*_*i*_ if *R*_*i*_=0 and *D*_*i*_=0 if *R*_*i*_=1. Model 2 describes observed outcomes as differing in expectation from untreated outcomes only through the receipt of treatment, where *β* is the treatment effect. The absence of a direct effect of *R* expresses the exclusion restriction, that the always-takers have the same mean outcome in both randomised arms. In fact, the model makes unnecessary and unused assumptions about the causal effect of treatment in always-takers: we return to this in the discussion. Model 2 implies for the observed outcome:

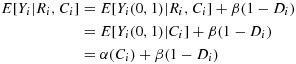
3.2
where *α*(*C*_*i*_) = *E*[*Y*_*i*_(0,1)|*C*_*i*_] represents the mean untreated outcome for individuals with compliance type *C*_*i*_: its dependence on *C*_*i*_ allows for indirect selection. Under this model, the causal effect of randomisation on outcome for compliers (*C*_*i*_=1) is

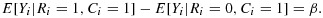
3.3
This is the difference in mean outcome between compliers randomised to treatment and compliers randomised to control. The parameter *β* represents the CACE, the average causal effect among the group of compliers.

Estimation of *β* is complicated by the fact that compliance type *C*_*i*_ is not observed for those randomised to treatment *R*_*i*_=1. A regression of outcome *Y*_*i*_ on randomisation *R*_*i*_ and compliance type *C*_*i*_ will not suffice because *C*_*i*_ is not fully observed. Instead, estimation can be achieved using either maximum likelihood or Bayesian methods. In Bayesian analysis, the unobserved compliance types are considered as missing data and estimated in the same way as the other parameters. Probability distributions for *β* and the other parameters are obtained and appropriate summary measures reported.

In trials with a repeatedly measured outcome and all-or-nothing compliance, a longitudinal version of model 3 may be fitted. If *Y*_*ij*_ is the outcome for individual *i* at visit *j*, then

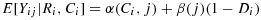
3.4
where *D*_*i*_ is defined as previously, and *α*(*C*_*i*_,*j*) = *E*[*Y*_*ij*_(0,1)|*C*_*i*_] represents the mean untreated outcome for individuals with compliance type *C*_*i*_ at visit *j*. Under this model, the causal effect of randomisation on outcome at visit *j* for compliers (*C*_*i*_=1) is

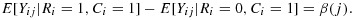
3.5
Thus, *β*(*j*) is the CACE at visit *j*. This model has previously been proposed and fitted by Yau and Little 19.

However, if treatment received is varying over time, the situation becomes more complicated. In our example, at a given visit *j*, those randomised to treatment would all have received treatment at the beginning of the trial, but those randomised to control will be a mixture of those who received treatment, one visit ago, two visits ago and so on up to *j* visits ago and will therefore be receiving different treatment effects at the current time. We now extend the CACE model to account for this by modelling the longitudinal data as follows.

## 4 Longitudinal complier average causal effect model

### 4.1 Complier average causal effect model for longitudinal compliance

As previously, let *R*_*i*_ be randomised arm (1 for treatment and 0 for control), and *Y*_*ij*_ be outcome for subject *i*∈1,...,*n*, visit *j*∈1,...,*m*. We redefine *D*_*i*_ as the last visit before surgical treatment (regarding baseline as visit 0): *D*_*i*_=0 if treatment was received between visits 0 and 1, *D*_*i*_=1 if treatment was received between visits 1 and 2 and so on, and *D*_*i*_=*m* if no treatment was received. *D*_*i*_ is therefore grouped, not actual, time of surgery. Let *D*_*i*_(*r*) be the potential value of *D*_*i*_ for subject *i* if randomised to treatment *r*. Let *Y*_*ij*_(*r*,*d*) be the potential outcome for subject *i* at visit *j* if randomised to *r* and receiving treatment just after visit *d*; we only model the treatment-free potential outcome *Y*_*ij*_(0,*m*). Again, we allow for indirect selection by allowing *C*_*i*_ to be associated with *Y*_*ij*_(0,*m*).

We assume those randomised to treatment receive surgery just after baseline, so that *D*_*i*_(1) = 0 for all *i*. Thus, *C*_*i*_, the latent compliance type for subject *i*, is again defined as *C*_*i*_=*D*_*i*_(0), the last visit before surgical treatment under randomisation to control. Now, *C*_*i*_ is categorical and is a summary of longitudinal compliance so is not dependent on time. The compliance types are principal strata in the terminology of Frangakis and Rubin 10. In particular, the principal strata with 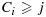 are the ‘compliers at visit j’, that is, those individuals who would receive treatment under randomisation to treatment but would receive no treatment up to visit *j* under randomisation to control.

We consider two causal models to specify the mean outcome, basing the treatment effect on (1) the number of visits since receiving treatment and (2) the number of days since receiving treatment. Both models describe the mean of *Y*_*ij*_−*Y*_*ij*_(0,*m*), which is the difference between an individual’s observed outcome and the same individual’s counterfactual outcome if they were randomised to control and never treated.

#### 4.1.1 Causal model using visits

The first model assumes equal spacing between visits and assumes that treatment occurs just after a visit:

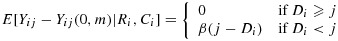
4.1
where *D*_*i*_=*C*_*i*_ if *R*_*i*_=0 and *D*_*i*_=0 if *R*_*i*_=1. Here, *β*(*k*), a function of *k* for *k*∈1,...,*m*, represents the causal effect of treatment on outcome measured *k* visits after treatment. We assume *β*(*k*) is equal across randomised arms: this identifying assumption is plausible in TARGET. This model implies for the observed data:

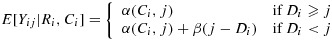
4.2
where *α*(*C*_*i*_,*j*) = *E*[*Y*_*ij*_(0,*m*)|*C*_*i*_] represents the mean untreated hearing loss for a patient with compliance type *C*_*i*_ at visit *j*; its dependence on *C*_*i*_ allows for indirect selection. The model embodies the exclusion restriction, because individuals of a given compliance type have the same mean untreated outcome, *α*(*C*_*i*_,*j*), in both randomised arms. Those randomised to control with 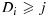 have not (yet) departed from their allocation (i.e. have not yet received any treatment), and so their expected outcome equals the mean untreated outcome *α*(*C*_*i*_,*j*). Those randomised to control with *D*_*i*_<*j* received treatment *j* − *D*_*i*_ visits ago, so their expected outcome is *α*(*C*_*i*_,*j*) + *β*(*j* − *D*_*i*_). Those randomised to treatment all have *D*_*i*_=0, so their expected outcome is *α*(*C*_*i*_,*j*) + *β*(*j*).

Under this model, the causal effect of randomisation on outcome at visit *j* for principal stratum *c* is

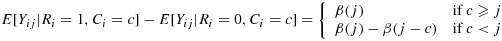
4.3
Thus, *β*(*j*) is the causal effect of randomisation on outcome at visit *j* for individuals in each of the principal strata with 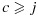. We therefore interpret *β*(*j*) as the average causal effect of randomisation on outcome at visit *j* among compliers at visit *j*.

#### 4.1.2 Causal model using days

We extend the aforementioned model to allow for unequal intervals between visits and to allow the causal effect of treatment to depend on the actual number of days since receiving treatment. Let the visits occur at *t*_1_,*t*_2_,…,*t*_*m*_ days after randomisation. The setup is the same as previously, but instead of using *D*_*i*_ to represent actual treatment, we now let *T*_*i*_ represent the time (in days) at which individual *i* first received treatment, or a value greater than *t*_*m*_ if treatment was never received. Compliance type is defined in terms of the potential treatment time under randomisation to control *T*_*i*_(0) as follows:

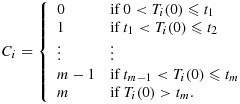

Let *β*(*j*) represent the treatment effect among compliers *t*_*j*_ days after receiving treatment, where *j* = 1,2,…,*m*. We assume a piecewise linear treatment effect between these times:

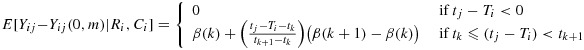
4.4
for *k* = 0,1,2,…,*m* − 1. This implies the observed data model

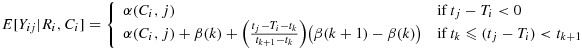
4.5
where *α*(*C*_*i*_,*j*) = *E*[*Y*_*ij*_(0,*m*)|*C*_*i*_] represents the mean untreated hearing loss for a patient with compliance type *C*_*i*_ at visit *j*, and *β*(*k*) is the average effect of randomisation on outcome at *t*_*k*_ days in the principal strata of compliers.

#### 4.1.3 Distributional model

In both models, the outcomes **Y**_*i*_=(*Y*_*i*1_,....,*Y*_*im*_) are assumed to have a multivariate normal distribution,

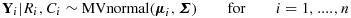

with means ***μ***_*i*_=(*μ*_*i*1_,...,*μ*_*im*_), where *μ*_*ij*_=*E*(*Y*_*ij*_|*R*_*i*_,*C*_*i*_) as given in 8 or 12, and ***Σ*** is an unstructured *m* × *m* covariance matrix. We also assume a saturated model for *C*_*i*_, that is, *p*(*C*_*i*_=*c*) = *γ*(*c*).

### 4.2 Assumptions

The aforementioned model makes several assumptions. The randomisation assumption implies that randomisation *R*_*i*_ is independent of pre-randomisation variables, including latent compliance type *C*_*i*_ and potential outcome *Y*_*i*_(0,1) 14. The stable unit treatment value assumption implies no interference between individuals, so that the compliance behaviour of one patient is not affected by the randomisation of other patients, and the potential outcome of one patient is not affected by the randomisation and compliance status of other patients. We also assume the causal model given by either 7 or 11, and that *β*(*k*) is equal across randomised arms.

### 4.3 Identification

We describe how the parameters *γ*(*c*), *β*(*j*) and *α*(*c*,*j*) (for *c* = 0,…,*m* and *j* = 1,…,*m*) are identified in the causal model using visits. A similar argument applies for the causal model using days.

1. Since *C*_*i*_ is observed if *R*_*i*_=0, *γ*(*c*) may be estimated using *γ*(*c*) = *P*(*C*_*i*_=*c*|*R*_*i*_=0).
2. Since E[*Y*_*ij*_|*R*_*i*_=1] = *β*(*j*) + E[*α*(*C*_*i*_,*j*)] and 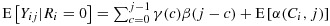, the ITT difference E[*Y*_*ij*_|*R*_*i*_=1] − E[*Y*_*ij*_|*R*_*i*_=0] may be expressed in terms of (*γ*(0),…,*γ*(*j* − 1)) and (*β*(1),…,*β*(*j*)). So the ITT difference at time *j* may be used recursively to estimate *β*(*j*).
3. Finally, we have E[*Y*_*ij*_|*C*_*i*_=*c*,*R*_*i*_=0] = *α*(*c*,*j*) if 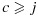 and =*α*(*c*,*j*) + *β*(*j* − *c*) if *c* < *j*. This may be used to estimate *α*(*c*,*j*).

The aforementioned procedure for estimating the *β*(*j*) is essentially the same as the instrumental variables procedure. However, the Bayesian procedure makes fuller use of the data.

### 4.4 Bayesian estimation

In Bayesian inference, we assume prior distributions for the parameters to be estimated and simulate the posterior distribution using the Gibbs sampler, treating the unobserved compliance types and missing outcomes as missing data. The CACE can only be indirectly estimated through the observation of mixtures of distributions. If compliance type is known for all units, inference of the causal estimands involves only data from the associated subpopulation with no mixture components. The first step of the data augmentation algorithm is to impute the missing compliance types by drawing them from their conditional distribution, a multinomial distribution, given observed data and current drawn values of *α*,*β*,*γ* and *Σ*. The second step is to draw values of parameters from the complete-data posterior distribution given current values of *C*_*i*_ and the observed values of *Y*_*i*_, *D*_*i*_ and *R*_*i*_. This involves drawing *α* and *β* from a multivariate normal distribution and *Σ* from an inverse Wishart distribution.

## 5 Application

We now apply the aforementioned models to data from the TARGET trial. To fit the models to the TARGET data, we make some simplifying assumptions to avoid creating too many compliance types. Non-compliance is assumed to occur in only one direction: those allocated to control can receive VT but not vice-versa. Some of those who received VT had the ventilation tube reinserted at a later time, but the reinsertions are also ignored here. In the TARGET trial, treatment may be received at any time, but we ignore its precise timing and define the compliance types as the last visit before which the individual would receive surgical treatment if randomised to control. *C* = 0 corresponds to those who would receive VT between visits 0 and 1 if they had been randomised to control, *C* = 1 corresponds to those who would receive VT between visits 1 and 2 if they had been randomised to control and so on. In this notation, *C* = 5 corresponds to those who would not have received VT at all, had they been randomised to control. The compliance types are unobserved in those randomised to treatment, but the model parameters may be estimated using Bayesian methods.

We analyse *n* = 248 individuals, 122 randomised to control (*R* = 0) and 126 randomised to VT (*R* = 1). There are five visits, so *j* = 1,2,3,4,5, and the corresponding number of days is taken to be *t*_1_=90,*t*_2_=180,*t*_3_=365,*t*_4_=550,*t*_5_=730.

Here, we assess the plausibility of assumptions made in Section 4.2. Treatment assignment was random in the TARGET trial, satisfying the randomisation assumption. The stable unit treatment value assumption (SUTVA) implies that the potential outcome for each individual does not depend on the treatment status of other individuals. This holds in TARGET because the hearing loss of one participant should not be affected by the treatment that other trial participants are receiving. The exclusion restriction means that treatment assignment is unrelated to potential outcomes given treatment received. This is plausible in TARGET because the outcome only depends on the time since receiving treatment and compliance type, rather than on randomisation. The monotonicity assumption implies that there are no defiers. In the TARGET example, most of those offered treatment took it up, so the assumption of no never-takers or defiers is plausible. The joint likelihood method assumes that the likelihood is correctly specified, namely normality of outcomes and a correctly specified covariance matrix.

### 5.1 Implementation

The aforementioned models were fitted using Markov chain Monte Carlo in WinBUGS 24 and were run for 100000 iterations. Diffuse normal distributions with mean zero and a large variance were used as prior distributions for the parameters *α*(*c*,*j*),*β*(*j*). An inverse Wishart distribution was used as a prior for ***Σ***.

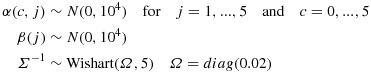

The posterior distribution was simulated, treating the unobserved compliance types and missing outcomes as missing data. We assume that the missing outcomes are missing at random 25, though other methods could be applied, as noted in the discussion. The simulations were run on two chains, which were initialised at different values near the maximum likelihood estimates. The first chain was initialised at *α*(*c*,*j*) = 20,*β*(*j*) =− 10 for all *candj* and *C*_*i*_=1 for all *i*. The second chain was initialised at *α*(*c*,*j*) = 10,*β*(*j*) =− 5 for all *c*,*j* and *C*_*i*_=1 for all *i*. Convergence was assessed using the Gelman–Rubin diagnostic 26 and history plots for each parameter. All of the model parameters, *α*(*c*,*j*),*β*(*j*),***Σ***, were mixing well, that is, the two chains were moving freely over the parameter space and appeared to have converged after about 10000 iterations.

The model using days since VT was implemented similarly. If there is a missing visit, we assume the number of missing days because receiving VT is equal to the scheduled number of days. This value is used to impute the missing *Y*_*ij*_. An alternative model for the missing days with an appropriate mean structure gave similar results.

### 5.2 Results

Table 2 gives the treatment effect estimates from an ITT analysis (model 1) and from the CACE model by visits since receiving VT (model 8). Under ITT, VT reduces hearing loss more than control, by 11.6dB with 95% CI (9.3 to 13.8)dB after one visit and by 5.6dB with 95% CI (3.1 to 8.1) after two visits. Under the CACE analysis, VT reduces hearing loss by 11.6 (9.2 to 14.0)dB compared to control after one visit and 7.2 (4.4 to 10.1)dB after two visits. The ITT analysis would be expected to give a conservative estimate of the treatment effect compared to the CACE model at visit 1, because the ITT analysis includes some patients in the control arm who have received VT one visit ago. In this case, the ITT and CACE estimates are similar for visit 1. For visits 3, 4 and 5, the sign of the ITT effect is positive, indicating a small but non-significant adverse effect of VT, whereas the CACE estimates are negative, indicating a small but non-significant beneficial effect of VT. This change is because at visit 3, for example, the control arm contains a mixture of patients, some of whom have received control and others who have received VT one, two or three visits ago.

Table 3 gives the treatment effect estimates from an ITT analysis and from the CACE model by days since receiving VT (model 12). We observe slightly larger treatment effects in both CACE and ITT analyses when taking into account actual days since receiving VT, rather than assuming equal spacing between visits. Qualitatively, both analyses agree that VT is significantly better for the first 6months after receiving VT. After 6months, no significant difference between VT and control is observed. By this time, a substantial proportion of those randomised to control have received VT, so the ITT analysis obscures a possible benefit of VT, whereas the CACE analysis indicates a non-significant beneficial effect.

**Table 3:** Estimates of treatment effect using number of days since VT.

Days since VT	ITT (95% CI)	CACE (95% CI)
90	−11.9(−14.3,−9.5)	−12.0(−14.3,−9.5)
180	−7.3(−9.8,−4.7)	−8.5(−11.2,−5.8)
365	−0.2(−2.9,2.7)	−2.5(−5.4,0.3)
550	1.0(−2.0,4.0)	−1.3(−4.7,2.0)
730	0.8(−1.5,3.2)	0.6(−2.1,3.7)

ITT (intention-to-treat) is the average effect of randomisation on observed outcome after *t* days.

CACE (complier average causal effect) is the average effect of randomisation on outcome at *t*_*k*_ days in the principal strata of compliers at *t*_*k*_ days (*β*(*k*) from model 12). VT, ventilation tubes.

**Figure 3:**
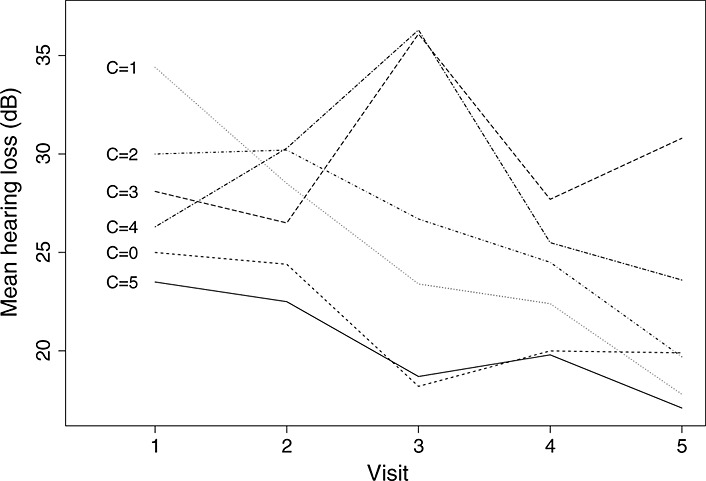
Estimates from model 8 of mean untreated outcome, *α*(*C*,*j*), by compliance type.

A graph of the estimates of *α*(*c*,*j*) from model 8, representing the untreated outcome over time for each compliance type, is given in Figure 3. Compliance type 0 has a relatively low hearing loss that gradually decreases over time. Compliance type 1 begins with a high hearing loss but decreases rapidly over time. Compliance type 2 has a relatively high hearing loss at the first two visits which then decreases. Compliance type 3 has moderate hearing loss at visits 1 and 2 then a very high hearing loss at visit 3 that decreases over the next two visits. Compliance type 4 starts with moderate hearing loss, increases to a high value at visit 3 then decreases. Finally, compliance type 5 begins with a low hearing loss that gradually decreases over time. The trajectories for those who receive VT immediately after baseline (C=0) and those who never receive VT under randomisation to control (C=5) are quite similar. This is consistent with early departures being due to discontentment with the allocation rather than poor outcomes.

### 5.3 Model checking

**Figure 4:**
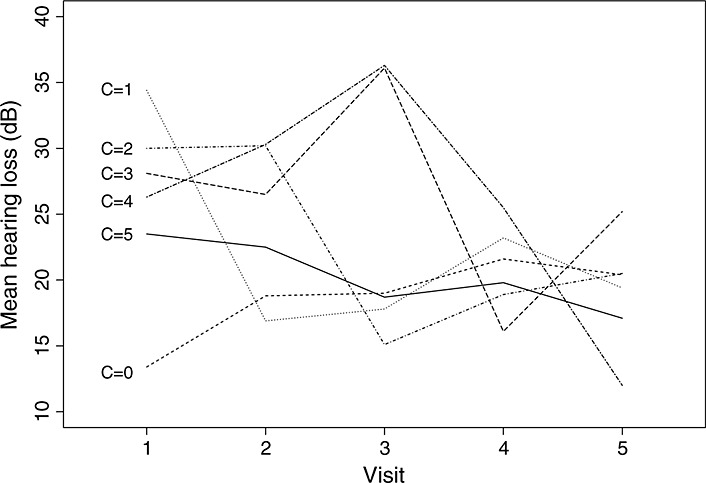
Fitted values from model 8 for control arm by compliance type.

**Figure 5:**
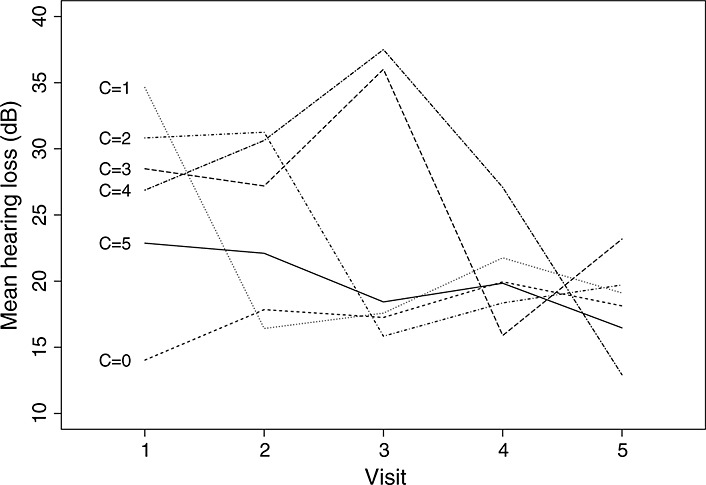
Mean outcome from model 8 for control arm by compliance type.

Plots of standardised residuals show that most lie within a reasonable range of about (−2.5,2.5). There are a few extreme residuals and these usually correspond to very high (>40 dB) outcomes. Exclusion of individuals with extreme residuals has little effect on the results. Plots of residuals versus fitted values show no distinguishable pattern. Comparison of the fitted values 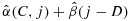 from model 8 (Figure 4) with the crude mean outcome in the control arm (Figure 5) suggests that the model makes fairly plausible assumptions.

Alternative options for *Ω* in the Wishart prior distribution were used, such as *Ω* = *diag*(0.001) and *Ω* = *diag*(1000), as well as non-diagonal matrices. These made little difference to estimates of the CACEs.

## 6 Simulation study

We performed a small simulation study to evaluate the performance of the proposed method and to compare it with the IV method in a data generating model loosely based on the TARGET results and the causal model using visits.

### 6.1 Data-generating model

We generated 1000 data sets of size 300 with *m* = 5 time points. We assumed equal randomisation (*p*(*R*_*i*_=1) = 0.5). The latent compliance types were distributed with *γ*(0) = *γ*(1) = *γ*(2) = *γ*(3) = *γ*(4) = 1/9, *γ*(5) = 4/9. The untreated mean outcomes were derived from TARGET as *α*(*c*,*j*) = 10 if *c* = 0,5; *α*(*c*,*j*) = 25 − *j* if *C* = 1,2; *α*(*c*,*j*) = 22 − 3|*j* − 3| if *C* = 3,4. The treatment effect was modelled as *β*(*j*) = 2(6 − *j*). Finally, *Y*_*ij*_ followed model 12 with uncorrelated *N*(0,8^2^) errors. There were no missing data.

### 6.2 Analyses

Model 8 was used. For comparison the IV, analysis was done, using dummy variables for treatment 1,…,5 visits ago as endogenous variables, the interaction of randomised group and time as instruments, and dummy variables for visits as covariates.

### 6.3 Results

**Figure 6:**
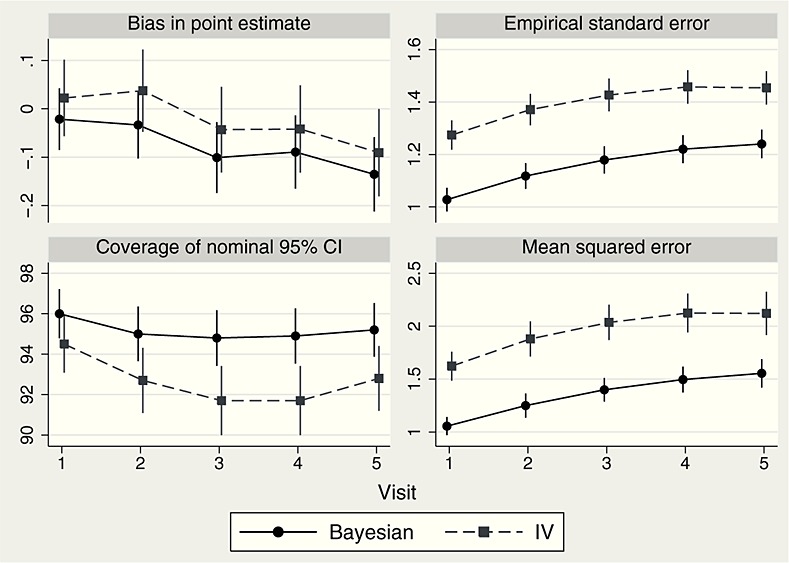
Results from the simulation study of Section 6. Monte Carlo error is expressed through 95% confidence intervals.

The results are summarised in Figure 6. Bias was small (magnitude ≤0.1 compared with treatment effects ranging from 10 to 2) and somewhat worse for the Bayesian method. However, the Bayesian method had a standard error 15–20% smaller than the IV method and hence a smaller mean squared error. Finally, the Bayesian method achieved 95% coverage near the correct 95% interval, while the IV method somewhat under-covered.

## 7 Conclusions and discussion

### 7.1 Conclusions

In randomised clinical trials in which a substantial proportion of patients departs from their randomised treatment, standard ITT analysis compares treatment policies but may obscure the treatment effect. Per-protocol analyses that compare those who adhere to their randomised allocation between randomised arms are commonly used, but these are subject to selection bias. Instead, randomisation-based estimates of efficacy may be employed. Given reasonable assumptions, the CACE model can provide estimates of the average causal effect of treatment among the group of compliers.

CACE models have previously been applied in simple situations where treatment is all-or-nothing and compliance is binary. We extended the CACE model to incorporate compliance that is changing over time by introducing categorical compliance types based on the time of receiving treatment. We specified a model for the conditional distribution of outcome given randomisation and compliance type and fitted this to obtain estimates of the causal effect of treatment at each visit. Full probability modelling enables model checking by comparing fitted values with the observed values, by checking for extreme residuals and by examining the plot of residuals versus fitted values.

We applied this model to data from the TARGET trial in which outcomes are measured over five time points, and departures from the control arm to surgical intervention could occur at any time. In this example, the CACE analyses generally gave larger estimates of treatment effect compared to the ITT effects. The ITT estimates are conservative in this case because at any given time, the control arm contains a mixture of people, some of whom are receiving the effect of surgery. Adjusting for the exact timing of the visit had little effect on the results.

The CACE model can provide a useful secondary analysis in addition to the primary ITT analysis. However, the average causal effect among compliers may not be a representative of the causal effect among the general population. In addition, the longitudinal CACE model is somewhat complex both conceptually and in terms of computation. Computation can be performed using either maximum likelihood or Bayesian methods, but software would be needed to make CACE estimation more accessible.

### 7.2 Discussion

In this paper, we focused mainly on contamination of the control arm, that is, those randomised to control receiving VT. The model could be extended to include non-receipt of VT in the VT arm by creating just one more compliance type, namely those who would not receive VT if randomised to it, and making a no-defiers (monotonicity) assumption that these individuals would also not have received VT if randomised to control.

We ignored baseline covariates such as trial centre and baseline hearing loss in the CACE models. Inclusion of covariates both in the outcome model and as predictors in the compliance model should improve efficiency but could make estimation more complex and is a topic for further work. Trials that are large enough to consider interactions between baseline and outcome allow identification of patients who benefit most. In TARGET, there was evidence that those who had worse hearing benefit more from treatment and such people were more likely to receive non-randomised surgery. This is one situation in which applying CACE analysis can be useful 3.

The model could be extended to incorporate a *k*-level treatment by including more compliance types, one for each level of each treatment. The mean outcome model may need to be changed to give a different treatment effect for each level of treatment. It would be possible to incorporate continuous compliance by modelling *α*(*c*,*j*), for example using a linear model. However, this may be too sensitive to the modelling assumptions.

We used the identifying assumption that the causal effect of VT was the same in both randomised arms. This might be false if randomisation to control modified the value of a subsequent VT. However, in TARGET, control involved watchful waiting, and very few cases received any active treatment, so the identifying assumption seems plausible. An alternative could be to allow the causal effect of VT on the outcome *k* visits later to be *β*(*k*) in the VT arm and *λβ*(*k*) in the control arm and to allow *λ* to vary over a range of values below 1.

A limitation of the models is that they assume missing data are missing at random. Much work has been carried out to adjust for both non-compliance and missing data, for example 27–30, and these methods could be incorporated into the models presented.

Models 2, 7 and 11 make stronger assumptions than are required. For example, model 2 unnecessarily equates *E*[*Y*_*i*_−*Y*_*i*_(0,1)|*R*_*i*_=1,*C*_*i*_=0], the causal effect of treatment in always-takers, to *E*[*Y*_*i*_−*Y*_*i*_(0,1)|*R*_*i*_=1,*C*_*i*_=1], the causal effect of treatment in compliers randomised to treatment. It would be sufficient to assume that *E*[*Y*_*i*_|*R*_*i*_=1,*C*_*i*_] − *E*[*Y*_*i*_|*R*_*i*_=0,*C*_*i*_] equals *β*(1 − *C*_*i*_), as is implied by (but does not imply) model 2.

An alternative to full probability modelling is to estimate the CACE using IV analysis 31. This is easier to implement than the full probability modelling method described here and avoids distributional assumptions but does not perform as well as the CACE in terms of operating characteristics such as bias and the width of 90% intervals 15,21.

## Footnotes

‡ In the main trial paper and elsewhere, the pre-randomisation visits are referred to as visits 1 and 2 and the post-randomisation visits are referred to as visits 3 to 7.
